# A case of preexcitation syndrome showing atypical atrioventricular nodal reentrant tachycardia and orthodromic atrioventricular reciprocating tachycardia with a bystander concealed nodoventricular/nodofascicular pathway

**DOI:** 10.1016/j.hrcr.2022.05.003

**Published:** 2022-05-13

**Authors:** Shushi Nishiwaki, Satoshi Shizuta, Munekazu Tanaka, Akihiro Komasa, Hirohiko Kohjitani, Takeshi Kimura

**Affiliations:** Department of Cardiovascular Medicine, Kyoto University Graduate School of Medicine, Kyoto, Japan

**Keywords:** Nodoventricular pathway, Nodofascicular pathway, Preexcitation syndrome, Atrioventricular nodal reentrant tachycardia, Atrioventricular reciprocating tachycardia, His-refractory ventricular extrastimulation

**Key Teaching Points**

• When concealed nodoventricular/nodofascicular (NV/NF) pathway connecting to slow pathway is present, His-refractory ventricular extrastimulation (VES) for atypical atrioventricular nodal reentrant tachycardia (AVNRT) is associated with reset of the tachycardia with delay and/or termination of the tachycardia without atrial capture.
• Also, in orthodromic atrioventricular reciprocating tachycardia, His-refractory VES can reset the tachycardia with delay when concealed NV/NF pathway connecting to slow pathway is present and the slow pathway is acting as the antegrade limb of the tachycardia circuit.
• Even when the effective refractory period of the bystander antegrade accessory pathway is far shorter than the cycle length of AVNRT, bystander preexcitation can continuously disappear during the tachycardia owing to a linking phenomenon.

## Introduction

The concealed nodoventricular/nodofascicular (NV/NF) pathways are known as retrograde bypass tracts connecting right ventricular muscle/right bundle branch and atrioventricular node. Most concealed NV/NF pathways connect to slow pathways (SPs). Although the concealed NV/NF pathway sometimes causes orthodromic reciprocating tachycardia (ORT) acting as a part of the reentry circuit, it is mostly a bystander conduction tract observed in patients with atrioventricular nodal reentrant tachycardia (AVNRT).^1^, ^2^, ^3^, ^4^, ^5^, ^6^, ^7^ However, orthodromic atrioventricular reciprocating tachycardia (AVRT) with bystander concealed NV/NF pathway in patients with accessory pathways (APs) has not been reported. Here we report a case of preexcitation syndrome showing atypical AVNRT and orthodromic AVRT with a bystander concealed NV/NF pathway.

## Case report

A 37-year-old man visited an outpatient clinic of a local physician because of recurrent episodes of loss of consciousness and convulsion. Wide QRS tachycardias were documented in Holter monitoring (Supplemental Figure 1A), and he was referred to our hospital. A 12-lead electrocardiogram showed delta waves suggesting posteroseptal AP (Supplemental Figure 1B). Echocardiography showed no structural heart disease. He underwent an electrophysiological study (EPS) followed by catheter ablation. Decapolar electrode catheters were placed at the His region and in the coronary sinus (CS). Quadripolar electrode catheters were placed at the high right atrium and right ventricular apex (RVA) (Supplemental Figure 2). The baseline A-H interval was 76 ms, and the H-V interval was 14 ms with preexcitation. In programmed single atrial extrastimulation (AES), AV conduction initially showed no decremental property with preexcitation, but after disappearance of preexcitation, decremental property and AV jump were observed. The effective refractory period (ERP) of the antegrade AP was 290 ms. In programmed single ventricular extrastimulation (VES), VA conduction initially showed no decremental property with earliest atrial activation site (EAAS) at mid CS. Thereafter, decremental property was observed with EAAS at His. Thus, there were at least 2 retrograde conductions through posteroseptal AP and fast pathway (FP), but we could not identify retrograde SP by single VES, presumably because of short ERP of the retrograde FP and intraventricular decremental conduction. The ERP of the retrograde AP was 280 ms.

**Figure 1:**
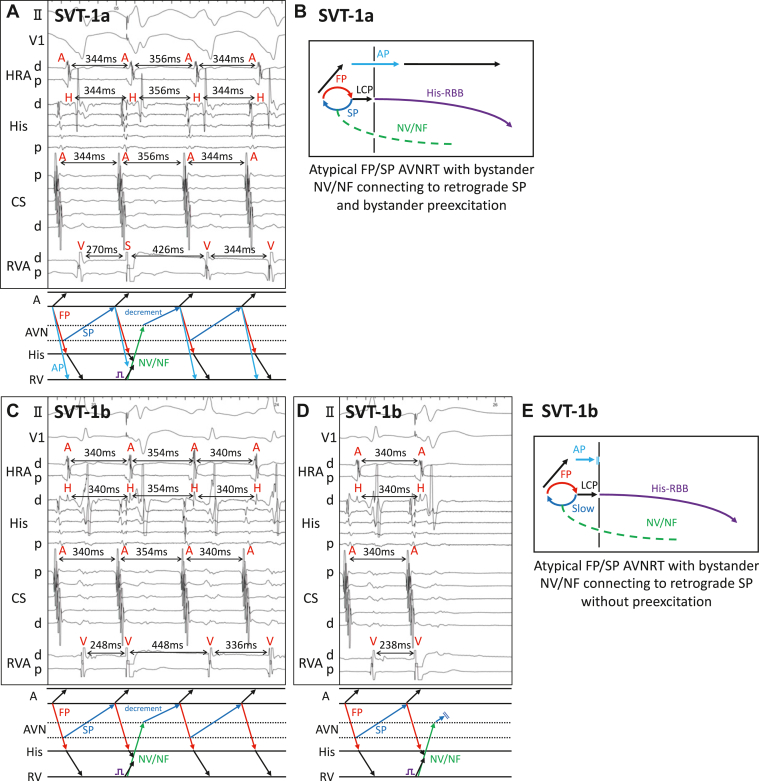
Responses to His-refractory ventricular extrastimulation (VES) in supraventricular tachycardia (SVT)-1a and SVT-1b. **A:** His-refractory VES for SVT-1a reset the tachycardia with delay of subsequent His and atrial electrograms. **B:** Diagram of SVT-1a. **C:** His-refractory VES for SVT-1b reset the tachycardia with delay of subsequent His and atrial electrograms. **D:** Earlier His-refractory VES for SVT-1b terminated the tachycardia without atrial capture. **E:** Diagram of SVT-1b. AP = accessory pathway; AVN = atrioventricular node; AVNRT = atrioventricular nodal reentrant tachycardia; CS = coronary sinus; FP = fast pathway; HRA = high right atrium; LCP = lower common pathway; NF = nodofascicular; NV = nodoventricular; RBB = right bundle branch; RV = right ventricle; RVA = right ventricle apex; SP = slow pathway.

Under isoproterenol infusion, supraventricular tachycardia (SVT)-1a was induced by programmed double AES (Supplemental Figure 3). SVT-1a was wide QRS, long RP tachycardia with EAAS at the CS ostium. The activation sequence was A-H-V, indicating that antidromic AVRT was unlikely. Ventricular overdrive pacing (VOP) from the RVA showed a pseudo-A-A-V pattern with a long postpacing interval minus tachycardia cycle length (TCL) of 218 ms, suggesting that atrial tachycardia (AT), NV/NF ORT, and orthodromic AVRT with slow AP were all unlikely (Supplemental Figure 4A).^8^^,^^9^ Another VOP for SVT-1a with a shorter pacing cycle length (PCL) led to termination of the tachycardia. The total pacing prematurity, defined as N × [TCL − PCL] (N = number of stimuli needed to reset the atrium or to terminate the tachycardia), was 200 ms, far longer than the cutoff value of 125 ms, also suggesting that concealed NV/NF ORT and orthodromic AVRT with slow AP were both unlikely (Supplemental Figure 4B).^10^ From those findings, SVT-1a was diagnosed as atypical FP/SP AVNRT with bystander preexcitation. Interestingly, VES at the timing of the His-refractory period reset SVT-1a with delay of subsequent His and atrial electrograms (Figure 1A). If SVT-1a had been simply atypical FP/SP AVNRT with bystander preexcitation, the His-refractory VES could not have reset the tachycardia with delay, suggesting the presence of an additional retrograde pathway. The sequence of delayed atrial electrograms and A-H interval after the VES were not changed, indicating that SVT-1a was reset by conduction through concealed NV/NF connecting to the retrograde SP, followed by decremental conduction of the SP, which led to delayed His and atrial electrograms (Figure 1A). Thus, SVT-1a was diagnosed as atypical FP/SP AVNRT with bystander concealed NV/NF pathway connecting to retrograde SP and bystander preexcitation (Figure 1B).

We delivered a single AES from the high right atrium during SVT-1a, which converted SVT-1a to SVT-1b (Supplemental Figure 5). SVT-1b had the same TCL and atrial activation sequence as those of SVT-1a, and the only difference was no bystander preexcitation in SVT-1b. This indicated that the AP entered the ERP by the AES, followed by sustained antegrade conduction through the fast pathway without preexcitation, which is called a linking phenomenon (Supplemental Figure 5).^11^^,^^12^ SVT-1b was also induced by double AES with disappearance of preexcitation without AV jump. VES from RVA at the His-refractory period also reset SVT-1b with delay of subsequent His and atrial electrograms (Figure 1C), just as in SVT-1a. Earlier His-refractory VES terminated SVT-1b without atrial capture (Figure 1D). Thus, SVT-1b was diagnosed as atypical FP/SP AVNRT with bystander concealed NV/NF pathway connecting to retrograde SP without preexcitation (Figure 1E).

**Figure 2:**
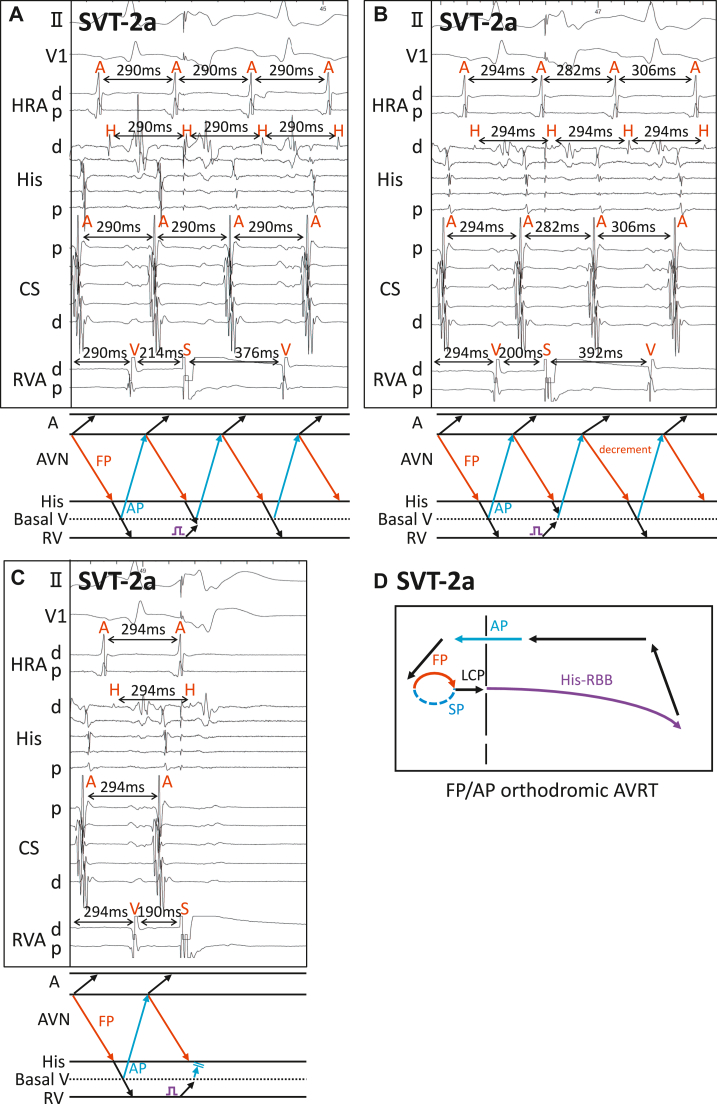
Responses to His-refractory ventricular extrastimulation (VES) in supraventricular tachycardia (SVT)-2a. **A:** In SVT-2a, VES at the timing of the His electrogram did not reset the tachycardia. **B:** Earlier His-refractory VES (His 14 ms) rest the tachycardia with advancement. **C:** Further earlier His-refractory VES (His 24 ms) terminated tachycardia without atrial capture. **D:** Diagram of SVT-2a. AVRT = atrioventricular reciprocating tachycardia; other abbreviations as in Figure 1.

SVT-2a was induced by programmed double AES with disappearance of preexcitation without AV jump. SVT-2a was narrow QRS, short RP tachycardia with EAAS at the mid CS, suggesting FP/AP orthodromic AVRT. VES from RVA at the timing of the His electrogram did not reset SVT-2a (Figure 2A), presumably because of ventricular conduction delay. Earlier VES at His-refractory period reset SVT-2a with the advancement of atrial electrograms without changes in their sequences (Figure 2B). Further earlier VES at His-refractory period terminated the tachycardia without atrial capture, indicating that AT was unlikely (Figure 2C). Thus, SVT-2a was diagnosed as FP/AP orthodromic AVRT (Figure 2D).

**Figure 3:**
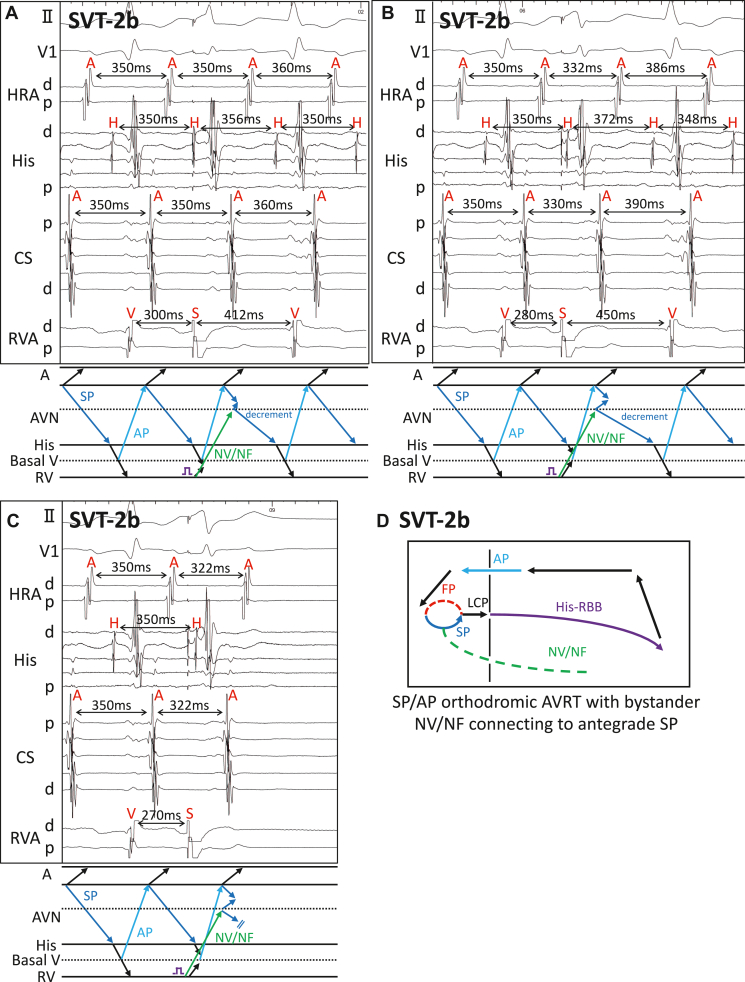
Responses to His-refractory ventricular extrastimulation (VES) in supraventricular tachycardia (SVT)-2b. **A:** In SVT-2b, VES at the timing of the His electrogram reset the tachycardia with delay of subsequent His. Although atrial electrograms immediately after VES were not reset, next atrial electrograms were delayed. **B:** Earlier His-refractory VES (His 20 ms) advanced the atrial electrograms immediately after the stimulation, but subsequent His electrogram was delayed owing to decremental conduction of the antegrade slow pathway (SP). **C:** Further earlier His-refractory VES (His 30 ms) advanced the atrial electrograms immediately after stimulation, leading to termination of the tachycardia. **D:** Diagram of SVT-2b. AVRT = atrioventricular reciprocating tachycardia; other abbreviations as in Figure 1.

SVT-2b was induced by programmed double AES with disappearance of preexcitation and AV jump. SVT-2b was narrow QRS, short RP tachycardia, and its EAAS was mid CS, suggesting SP/AP orthodromic AVRT. SVT-2b spontaneously terminated with AV block, suggesting that AT was unlikely. VES at the timing of the His electrogram reset SVT-2b with delay of subsequent His electrograms. It did not reset atrial electrograms immediately after the stimulation but delayed next atrial electrograms. This could be explained by the VES capturing concealed NV/NF pathway connecting to the antegrade SP, followed by decremental conduction of the SP (Figure 3A). Earlier VES at the His-refractory period reset SVT-2b with advancement of the atrial electrograms immediately after stimulation and delay of subsequent His electrograms (Figure 3B). Further earlier VES at the His-refractory period advanced subsequent atrial electrograms, leading to termination of the tachycardia (Figure 3C). The latter 2 phenomena of reset with advancement were considered that the VES captured atrium through AP, which is commonly observed in orthodromic AVRT. Thus, SVT-2b was diagnosed as SP/AP orthodromic AVRT with bystander concealed NV/NF pathway connecting to antegrade SP (Figure 3D).

From those results of the EPS, we targeted both the AP and SP in the ablation procedure. First, we targeted the posteroseptal AP, ablating left and right posterior septum as well as inside the CS, leading to complete elimination of antegrade and retrograde conduction of the AP. Subsequently, we performed anatomical SP ablation at the ostium of the CS. Many junctional beats were observed during the energy applications, and AV jump disappeared. Thereafter, no tachycardia was induced by programmed stimulation under isoproterenol infusion. The patient has been free from any symptoms for 6 months after the ablation procedure. Twelve-lead electrocardiography at 6 months showed no delta wave.

## Discussion

The present case was a rare case of preexcitation syndrome showing 4 types of SVT: 1a, atypical FP/SP AVNRT with bystander concealed NV/NF connecting to retrograde SP and bystander preexcitation; 1b, atypical FP/SP AVNRT with bystander concealed NV/NF connecting to retrograde SP without bystander preexcitation; 2a, FP/AP orthodromic AVRT; and 2b, SP/AP orthodromic AVRT with bystander concealed NV/NF pathway connecting to antegrade SP.

The VES at the His-refractory period is widely performed in the EPS of SVT to differentiate AVNRT and orthodromic AVRT. Typically, tachycardia is not reset by His-refractory VES in AVNRT, but is reset with advancement of subsequent atrial and His electrograms in orthodromic AVRT. However, when concealed NV/NF pathway connecting to SP is present in patients with atypical AVNRT, the His-refractory VES is associated with reset of the tachycardia with delay and/or termination of the tachycardia without atrial capture.^1^, ^2^, ^3^, ^4^, ^5^, ^6^, ^7^ The former was observed in SVT-1a and SVT-1b, and the latter was observed in SVT-1b. The reset of the tachycardia with delay occurs because the decremental conduction of the retrograde SP to which the concealed NV/NF pathway is connected exceeds the earliness of the VES. Termination of the tachycardia without atrial capture occurs because early capture of the retrograde SP through the concealed NV/NF pathway results in the block of the conduction to the atrium through the SP owing to ERP.

Among 10 patients from 7 previous reports of atypical AVNRT with bystander NV/NF pathways, the His-refractory VES was associated with reset of the tachycardia, with delay in 4 patients (40%) and termination of the tachycardia without atrial capture in 7 patients (70%) (Supplemental Table 1).^1^, ^2^, ^3^, ^4^, ^5^, ^6^, ^7^ Either phenomenon was observed in all patients.

His-refractory VES is affected by concealed NV/NF pathway only when SP is a part of the reentry circuit. In SVT-2a of the present case, which was FP/AP orthodromic AVRT, SP was not involved in the reentry circuit. Therefore, the His-refractory VES just showed reset of the tachycardia through AP advancing subsequent atrial electrograms, which is commonly observed in orthodromic AVRT. In SVT-2b, which was SP/AP orthodromic AVRT, the SP was the antegrade limb of the reentry circuit. The His-refractory VES reset the tachycardia with delay of subsequent His electrogram and following atrial electrograms (Figure 3A). The reason why the VES captured the concealed NV/NF pathway but not the AP was probably because the concealed NV/NF pathway was located nearer to the RVA stimulation site than the AP. The same phenomenon can be observed in SP/FP typical AVNRT with bystander concealed NV/NF pathway. Sekihara and colleagues^13^ reported reset phenomenon with delay after His-refractory VES in patients with typical SP/FP AVNRT with bystander concealed NV/NF pathway, just as in SVT-2b of the present case.

There were several limitations in the present case report. First, in the VOP for SVT-1a shown in Supplemental Figure 4A, the atrium was entrained only for 1 beat by the last pacing. We should have repeatedly performed VOP for SVT-1a until the atrium was constantly entrained for several beats without termination of the tachycardia. Second, we performed VOP maneuvers only for SVT1a, but not for other SVTs. However, we believe that the diagnoses of all 4 SVTs were robust, because those were made comprehensively based on the EPS findings, including induction patterns of the tachycardias, TCLs, VA intervals, EAASs, QRS widths and morphologies, and responses to VOP and His-refractory VES during the tachycardias.

To the best of our knowledge, this is the first report of preexcitation syndrome with atypical AVNRT and orthodromic AVRT with a bystander concealed NV/NF pathway connected to SP conducting both antegradely and retrogradely.

## Conclusion

VES at the His-refractory period in the EPS of SVT is associated with reset of the tachycardia with delay and/or termination of tachycardia without atrial capture when bystander concealed NV/NF pathway connecting to SP is present and the SP is acting as a part of the reentry circuit, both in AVNRT and in orthodromic AVRT.
