# Supplementary material for: A case of preexcitation syndrome showing atypical atrioventricular nodal reentrant tachycardia and orthodromic atrioventricular reciprocating tachycardia with a bystander concealed nodoventricular/nodofascicular pathway
Source: HeartRhythm Case Rep. 2022 May 13;8(7):529–34. doi: 10.1016/j.hrcr.2022.05.003 (PMC9289061; doi:10.1016/j.hrcr.2022.05.003)
Supplement: Supplemental Figures 1–5 and Supplemental Table 1 [file mmc1.docx]

**Supplemental Materials**

**Supplemental Appendix**

Supplemental Table ----------------------------------------------------------------- Page 2

Supplemental Figure 1 ----------------------------------------------------------------- Page 3

Supplemental Figure 2 ----------------------------------------------------------------- Page 4

Supplemental Figure 3 ----------------------------------------------------------------- Page 5

Supplemental Figure 4 ----------------------------------------------------------------- Page 6

Supplemental Figure 5 ----------------------------------------------------------------- Page 7

**Supplemental Table. Summary of responses to the His refractory VES in atypical AVNRT with bystander NV/NF pathway**

| **Number** | **Tachycardia** | **Details of NV/NF pathway** | **Response to His refractory VES** | | |
| --- | --- | --- | --- | --- | --- |
|  |  |  | **Reset with advancement** | **Reset with delay** | **Termination without atrial capture** |
| 1 | Atypical AVNRT with bystander NV/NF pathway | NF^1^ | (-) | (-) | (+) |
| 2 |  | NV/NF^2^ | (-) | (-) | (+) |
| 3 |  | NV/NF^3^ | (-) | (+) | (+) |
| 4 |  | NV/NF^4^ | (-) | (-) | (+) |
| 5 |  | NF^5^ | (-) | (-) | (+) |
| 6 |  | NF^6^ | (-) | (+) | (-) |
| 7 |  | NV^7^ | (-) | (-) | (+) |
| 8 |  | NV^7^ | (-) | (+) | (-) |
| 9 |  | NV^7^ | (-) | (-) | (+) |
| 10 |  | NV/NF^7^ | (-) | (+) | (-) |
| Current |  | NV/NF | (-) | (+) | (+) |

VES, ventricular extra stimulation; AVNRT, atrioventricular nodal reentrant tachycardia; NV, nodoventricular; NF, nodofascicular.

**Supplemental Figure 1.**

1. Clinical tachycardia documented in Holter monitoring.
2. Twelve leads electrocardiogram during sinus rhythm on hospital admission.

**Supplemental Figure 2.**

The fluoroscopic images of the placement of electrode catheters at high right atrium, coronary sinus, His, and right ventricular apex.

**Supplemental Figure 3. Induction of SVT-1a**

Double atrial extra stimulation induced SVT-1a. After the second extra stimulation, preexcitation disappeared followed by two reentries with EAAS at the mid of CS, leading to initiation of SVT-1a with V-A-A-V pattern.

HRA, high right atrium; CS, coronary sinus; RVA, right ventricle apex; AVN, atrioventricular node; LCP, lower common pathway; RV, right ventricle; AP, accessory pathway; FP, fast pathway; SP, slow pathway; SVT, supraventricular tachycardia; EAAS, earliest atrial activation site.

**Supplemental Figure 4. Ventricular overdrive pacing from RVA in SVT-1a**

1. After cessation of ventricular overdrive pacing with a PCL of 330ms, SVT-1a resumed with pseudo A-A-V pattern. PPI -TCL was 570-352 =218ms, far longer than 125ms. Also, total pacing prematurity defined as N x [TCL-PCL] (N; number of stimuli needed to reset the atrium or to terminate the tachycardia) was 9 x (352-330) =198ms, far longer than the cutoff value of 125ms.

1. Ventricular overdrive pacing with a shorter pacing cycle length of 320ms terminated SVT-1a. Total pacing prematurity was 5 x (360-320) =200ms, far longer than the cutoff value of 125ms

RVA, right ventricle apex; SVT, supraventricular tachycardia; HRA, high right atrium; CS, coronary sinus; PCL, pacing cycle length; PPI, post-pacing interval; TCL, tachycardia cycle length.

**Supplemental Figure 5. Conversion from SVT-1a to SVT-1b by single atrial stimulation**

Single atrial stimulation converted SVT-1a to SVT-1b. Disappearance preexcitation was considered due to a linking phenomenon.

SVT, supraventricular tachycardia; HRA, high right atrium; CS, coronary sinus; RVA, right ventricle apex.
